# Managing Intractable Natural Resource Conflicts: Exploring Possibilities and Conditions for Reframing in a Mine Establishment Conflict in Northern Sweden

**DOI:** 10.1007/s00267-023-01838-5

**Published:** 2023-06-08

**Authors:** Andreas Johansson

**Affiliations:** Unit of Political Science, Luleå University of Technology, 97187 Luleå, Sweden

**Keywords:** Natural resource management, Intractable conflicts, Conflict management, Reframing, Interpretive policy analysis, Frame analysis

## Abstract

Natural resource management (NRM) increasingly relies on communicative measures to enable reframing in intractable conflicts. Reframing occurs when disputants change their perceptions of a conflict situation, and/or their preferences for dealing with it. However, the types of reframing possible, and the conditions under which they can occur, remain unclear. Through an inductive and longitudinal analysis of a mine establishment conflict in northern Sweden this paper explores to what extent, how, and under what conditions reframing can occur in intractable NRM conflicts. The findings reveal the difficulty in achieving consensus-oriented reframing. Despite multiple dispute resolution efforts, the disputants’ perceptions and preferences became increasingly polarized. Nonetheless, the results suggest that it is possible to enable reframing to the extent that all disputants can understand and accept each other’s different perceptions and positions, i.e., meta-consensus. Meta-consensus hinges on neutral, inclusive, equal, and deliberative intergroup communication. However, the results show that intergroup communication and reframing are significantly informed by institutional and other contextual factors. For example, when implemented within the formal governance system in the investigated case, intergroup communication lagged in quality and did not contribute to meta-consensus. Moreover, the results show that reframing is strongly influenced by the nature of the disputed issues, actors’ group commitments, and the governance system’s distribution of power to the actors. Based on these findings, it is argued that more efforts should focus on how governance systems can be configurated so that high-quality intergroup communication and meta-consensus can be enabled and inform decision making in intractable NRM conflicts.

## Introduction

The viability of our democratic systems largely depends on their capability to reach legitimate decisions in the face of public conflicts (Rein and Schön 1996). However, in the context of natural resource management (NRM), increasing competition for the world’s natural resources are spurring complex, protracted, and value-based conflicts (concerning e.g., water, land, forests, energy, fisheries, and minerals) which seemingly defy all efforts to develop such decisions (Conde 2017; Nie 2003). Conflicts sharing these characteristics are often referred to as “intractable” (Asah et al. 2012).

How to “best” manage intractable conflicts is broadly debated, with different scholars offering varying solutions (e.g., Gray 2003; Mouffe 1999; Rein and Schön 1996). In recent NRM practice, there has been a growing emphasis on utilizing various forms of intergroup communicative processes, including deliberation (Bäckstrand et al. 2010; Montambeault et al. 2020), collaborative planning (Ansell and Gash 2007; Fisher et al. 2020), consensus building (Susskind et al. 2020), and multi-actor dialogues (Cuppen et al. 2010; Schultz et al. 2018). Despite significant variation, the implementation of these processes is often guided by the same belief: that inclusive, equitable, and reasoned discussions about contentious issues have the potential to facilitate a “reframing” of conflicting perspectives, ultimately leading to mutually accepted agreements or consensus (Bächtiger et al. 2018; Bäckstrand et al. 2010; Curato et al. 2017). Reframing occurs when disputants alter their perceptions of a conflict situation and/or their preferences for dealing with it (Gray 2003; Schön and Rein 1994). However, despite the increasing reliance on communicative reframing efforts in NRM practice, the evidence regarding how, to what extent, and under what conditions reframing can occur in intractable NRM conflicts is inconsistent, and the potential for managing conflicts through intergroup communication are therefore unclear (Davies et al. 2016; Feist et al. 2020).

Research exploring the potential of intergroup communication to facilitate reframing in intractable NRM conflicts has grown considerably over the years (Hossu et al. 2018; Putnam 2010). The majority of this research has concentrated on the prospects of achieving consensus, defined as a mutually accepted agreement on courses of decision and/or action (Hossu et al. 2018; Putnam 2010). There is a broad agreement in the literature that consensus-oriented reframing is most effectively facilitated through communicative processes that are deliberative (characterized by mutual reason-giving and listening), inclusive (including all perspectives present in the conflict), equal (considering all perspectives and giving equal opportunity to influence), non-coercive, guided by an impartial and skilled mediator, and characterized by clear, flexible, and mutually agreed rules, agenda, and objectives (Buchecker et al. 2021; Emerson et al. 2009; Schön and Rein 1994; Susskind et al. 2020). However, the findings regarding the outcomes of these processes are inconsistent. While some studies suggest that consensus is possible (Asah et al. 2012; Buchecker et al. 2021; Burgess and Burgess 2006) others question its attainability (Kahan et al. 2011; Terpstra et al. 2009) or find no signs of consensus-oriented reframing (Johansson et al. 2022).

To explain the variable results, research on the role of conditions outside the communication process has been requested (Baker and Chapin III 2018; Bächtiger and Parkinson 2019; Raitio 2013). Recent research highlights a range of contextual factors that facilitate intergroup communication in conflict. These include e.g., community relations that make actors feel comfortable talking and listening to each other (Baker and Chapin III 2018; Johansson et al. 2022); external events/shocks, such as environmental disasters, that awaken the disputants to the risks of intractability (Montambeault et al. 2020); and structures of institutions or rules that ensure that all disputants are provided with capabilities (e.g., rights and resources) for equitable participation and influence in communicative processes and decision making (Arai et al. 2021; Baker and Chapin III 2018; Raitio 2013). However, it is still unclear how such contextual factors influence the prospects for reframing, and research is yet to specify what types of reframing can be realistically enabled under different conditions of conflict (Kuyper 2018; Montambeault et al. 2020).

Accordingly, more research on the interrelationship between context, intergroup communication, and reframing is needed. As studies have just begun unraveling the contextual factors that influence reframing, inductive studies are motivated. Moreover, as reframing is a process that occurs over time, as conflicts develop and change, longitudinal approaches are suitable (Desrosiers 2012).

The aim of the present study is to explore to what extent, how, and under what conditions, reframing can occur in intractable NRM conflicts. In response to the aforementioned research gaps, an inductive and longitudinal approach is employed, focusing on the ongoing intractable conflict surrounding a mine establishment in Jokkmokk, Sweden. Specifically, the study employs interpretive process tracing, involving two key steps. Firstly, the conflict and the perspectives (or frames) of the disputing actors are traced over a span of five years, enabling the extent and nature of reframing over the course of the conflict to be identified. Secondly, in-depth interviews are used to delve into the factors and mechanisms that enabled and inhibited reframing among the actors.

The investigated conflict has persisted for over a decade and stands out as one of the most complex, long-lasting, and controversial mining conflicts in Sweden (Beland Lindahl et al. 2018). It revolves around a contentious regulatory framework and policy/permit process, and has sparked protests and civil disobedience to an extent rarely witnessed before in Sweden (Wilson and Allard 2022). Moreover, the case reflects the broader trends observed in NRM in that it is situated in a policy context of increasing conflicts (Fjellborg et al. 2022) to which the State increasingly respond with intergroup communicative measures to foster consensus-oriented reframing (Johansson et al. 2022; Pölönen et al. 2020). Hence, the case offers rich opportunities to advance inductive knowledge regarding the possibilities and conditions for reframing in intractable NRM conflicts; knowledge which can serve as a valuable resource for academics and policymakers, as well as for practitioners involved in the management of conflicts.

## Theory

### Reframing

The study explores reframing following the frame analytical approach developed by Schön and Rein (1994) and van Hulst and Yanow (2016). Frame analysis in this approach is a theory and method for conceptualizing and exploring action formations and actions in intractable policy conflicts based on a constructivist understanding of the social world. In this approach, actions are conceptualized as products of frames which are diagnostic (including perceptions of a situation) and prescriptive (including preferences for acting in, and dealing with, a situation) meaning constructions built up through the interactions (including both communication and practices) actors engage in when they grapple with various situations from their particular concerns and preconceptions (Schön and Rein 1994; van Hulst and Yanow 2016). This approach departs from perspectives in social movement research that view frames and reframing as strategic processes employed to attain predetermined objectives (see van Hulst and Yanow 2016 for a review of the differences). While such perspectives are valuable for investigating the strategic dimensions of meaning-making, they are poorly aligned with the objective of this study, which seeks to explore reframing beyond strategic processes.

Specifically, a frame selects certain elements (related to the issues, actors, relations, institutions, and the policy process) from a situation while ignoring others; assigns particular names and interpretations to the selected elements, employing metaphors, analogies, definitions, categorizations, and value-laden language; and organizes these elements into a coherent and graspable storyline that elucidates the ongoing situation and outlines necessary actions (Schön and Rein 1994; van Hulst and Yanow 2016).

Conflicting frames are commonly found in the context of NRM, prioritizing different socio-economic and environmental elements, placing importance on different scales, and/or giving the same elements radically different interpretations (Asah et al. 2012; Burgess and Burgess 2006). When actors in the same policy context develop and stick with frames that are incompatible to the extent that they prevent any form of agreement or legitimate decisions from being worked out, intractable conflicts typically occur (Gray 2003; Rein and Schön 1996).

Reframing, which is the focus of the present study, is the process of changing frames. It occurs when actors change how they perceive a specific situation (including their understanding of other actors and their respective frames in the situation) and/or their preferences for dealing with it (Gray 2003; Schön and Rein 1994). Reframing can entail a wide range of changes in the actors’ frames, which can serve to both escalate and de-escalate conflict. Most communicative approaches for managing conflict strive for consensus, minimally defined as mutually accepted agreements on courses of decision and/or action (Asah et al. 2012; Buchecker et al. 2021; Susskind et al. 1999). Consensus applies to a situation where the disputing actors’ frames have been changed to the extent that a common preference on how to deal with the situation is prescribed by all actors, albeit for different reasons.

Some scholars are critical of consensus as a guiding principle. They believe e.g., that, by appealing to unity in the face of pluralism, it privileges dominant frames held by powerful groups at the expense of marginalized and dissenting frames (see, e.g., Mouffe 1999). Accordingly, meta-consensus has been introduced as an alternative outcome (Dryzek and Niemeyer 2006). Meta-consensus applies to a situation in which the present frames are mutually understood and accepted by all actors despite disagreement. In contrast to consensus, meta-consensus does not require actors to converge around a mutually accepted preference. However, it does require the actors involved to develop an understanding of each other’s frames and, when necessary, adapt their frames in ways that ensure acceptability among their counterparts. As a result, meta-consensus fosters a mutually accepted plurality of frames. It responds to intractability by promoting respect between disputants and their respective frames. Additionally, by clarifying the conflict and its dividing lines, meta-consensus facilitates efforts to reach agreements when feasible/possible and make fair choices or trade-offs when they are not (Curato et al. 2017; Dryzek and Niemeyer 2006).

### Conditions Influencing Reframing

A challenge for the management of intractable conflicts is that reframing is a difficult process (Rein and Schön 1996). Frames represent actors’ ways of mastering situations and are often developed through great effort. Therefore, actors are often reluctant to question and change their frames (Rein and Schön 1996). Institutions (both norms and formal rules) in the actors’ social settings can also work against reframing (Perri 6 2005; Schön and Rein 1994). Through mechanisms such as internalization, socialization, sanctions, rewards, and social policing institutions reproduce particular sets of understandings and actions while preventing alternatives that challenge them (Schön and Rein 1994; Weber and Glynn 2006). Hence, the actors’ commitments and accountability to rules and norms in their social settings set limits which may require institutional changes to overcome.

Despite the challenges, reframing can occur, even in intractable conflicts (Burgess and Burgess 2006; Schön and Rein 1994). To facilitate this process, it is commonly theorized that actors must adopt a “frame-reflective” mode, characterized by engaging in reflective distancing, redirecting thoughts towards the frames, and being receptive to alternative meanings and actions (Schön and Rein 1994). When this mode is activated, a window for reframing emerges.

According to frame theory, intergroup communication plays a crucial role in facilitating frame reflection. Specifically, intergroup communication allows actors to engage in reciprocal inquiries about the alternative frames that exist in a conflict, which may not happen in the absence of such communication (Gray 2003; Putnam 2010; Rein and Schön 1996). Extensive efforts have been devoted to identifying the optimal design of the communicative process to effectively facilitate frame reflection and consensus-oriented reframing. The general recommendation is a process that is deliberative (characterized by a mutual give-and-take of frames), inclusive (including all frames present in the conflict), equal (considering all frames and giving equal opportunity to influence), non-coercive, guided by an impartial and skilled mediator, and characterized by clear, flexible, and mutually agreed rules, agenda, and objectives (Buchecker et al. 2021; Emerson et al. 2009; Susskind et al. 2020).

Frame reflection can also be prompted by various contextual changes. Specifically, contextual changes are believed to induce frame reflection by generating inconsistencies between the conflict situation and the frames which the actors employ to make sense of it (Laws and Rein 2003). When such inconsistencies arise, various elements (e.g., actors, institutions, or material structures) in the situation will begin resisting, talking back, defying the actors’ expectations, or even punishing the actors, to which they must respond with frame reflection and reframing to figure out what is going on and regain control (Laws and Rein 2003).

Intergroup communication and contextual changes are also interrelated. For example, when frame inconsistences occur, engagement with alternative frames is often necessary, making reciprocal communication more probable (Laws and Rein 2003). Research has also found that meaningful intergroup communication sometimes hinges on contextual changes that level power imbalances and incentivize disputants to engage with each other (Arai et al. 2021). These can include external shocks, such as environmental disasters, that awaken the disputants to the risks and uncertainties of non-collaboration (Montambeault et al. 2020). They can also include regulatory changes that ensure that all actors have the opportunity (e.g., rights and resources) to participate and influence in intergroup communication and decision making on an equitable basis (Raitio 2013). Furthermore, shifts in community relations may also be needed for actors to feel comfortable talking and listening to each other (Baker and Chapin III 2018).

Following the presented theory, this study explores reframing as changes in the disputing actors’ frames, which include their perceptions of the situation and their preferences for dealing with it. To advance the current knowledge about the factors influencing reframing, an inductive approach is used. This entails identifying and analyzing factors as they are revealed in the data, allowing for an open exploration of the factors that enable or inhibit reframing over the course of the conflict. Additionally, this approach facilitates the discovery of previously undiscovered factors. The methodological aspects of the study are outlined in the following.

## Research Design and Methods

### Research Design and Case

The study utilizes a longitudinal single case study design (Lewis and Nicholls 2014). The selected case is an ongoing intractable conflict over the establishment of an open-pit iron mine in Kallak, Jokkmokk, a traditionally resource-dependent, peripheral, and sparsely populated municipality in northern Sweden. The municipality is located near areas of documented cultural and nature conservation value (e.g., the United Nations Educational, Scientific, and Cultural Organization (UNESCO) World Heritage Site Laponia^1^). A large proportion of its population are Indigenous Sami, some of whom have reindeer husbandry rights.^2^ The conflict was sparked when the British company Beowulf Mining PLC (hereafter referred to as “the company”) was granted an exploration permit in 2006. Since then, several non-violent and violent protests and blockades have been staged, and actors sharing highly conflicting frames are still mobilizing on different sides (Beland Lindahl et al. 2018; Wilson and Allard 2022). In addition, the formal mine permit process, regulated by the Swedish Minerals Act (1991:45)^3^ and Environmental Code (1998:808), has been described as the most controversial and contentious permit process in modern Swedish history, not least because of political and legal disputes concerning Indigenous rights and the effects of mining on the local Sami reindeer herding communities (RHCs) (Wilson and Allard 2022). During the course of the conflict, a range of intergroup communicative processes have been organized, including corporate consultations, dialogue meetings, information meetings, and citizens’ dialogues (see result section). In sum, the case offers rich opportunities to inductively develop knowledge about the possibilities and conditions for reframing in intractable conflicts.

### Method, Material, and Analytical Techniques

The study utilizes the method of interpretive process tracing. This is a method for studying how processes unfolding over time produce particular outcomes (Norman 2015). It is an inductive method through which the factors causing particular outcomes are chiseled out by carefully reconstructing the context and sequence of events leading to the outcomes, and by exploring how the actors involved oriented themselves and assign meaning to these. The method is operationalized in two steps, reflecting the different parts of the aim. Firstly, based on document and frame analysis, the conflict and the actors’ frames are traced for the period January 2015 to January 2021^4^ (the study period) to identify key developments and events in the conflict, as well as to establish to what extent, and how, the actors reframed over time. Secondly, to determine which factors enabled or inhibited reframing, in-depth interviews are used to explore how the actors assign meaning to their reframing or frame stability and the developments/events in the conflict.

The empirical material used consists of: media reports from Swedish online and print media (>600 items) collected from the Retriever media archive using the search terms “Kallak/Gállok AND mine” OR “Kallak/Gállok AND conflict”; publicly available documents (e.g., newsletters and websites) created by the actors; public documentation from the permit process collected from the authorities; regulatory documents including relevant legislation (the Minerals Act (1991:45) and the Environmental Code (1998:808)); legislative changes and preparatory work collected from the Swedish Government Office’s legal databases; and longitudinal, in-depth interviews with disputing organized actors, conducted at the beginning and end of the study period (see Table 1). Actors included (15 in total) are the company, as well as locally organized actors that have taken some form of action in the conflict. The actors participated in the role as representatives of their organization, and were identified through early field work, document analysis, and a snowballing technique (Ritchie et al. 2014).

**Table 1:** Actors and interviews

Actors	First interview	Second interview
Jåhkågasska reindeer-herding community	2015-02-24	2020-12-04
Sirges reindeer-herding community	2015-02-25	2020-10-06
No Mines in Jokkmokk (resistance movement against mine establishment)	2015-03-09	2020-09-25
Swedish Society for Nature Conservation in Jokkmokk	2015-02-25	2020-10-08
Björkholmen Village Association	2015-01-30	2020-09-17
Sami Wellbeing (local political party)	2015-01-28	2020-10-01
Strukturum i Jokkmokk AB (organization for local business development)	2015-02-03	2020-09-22
Jokkmokk Forest Common (landowner association)	2014-11-05	2020-12-10
Jokkmokk Business Association	2015-04-29	2020-09-25
Destination Jokkmokk (local tourism business)	2015-02-25	2020-12-03
Jokkmokk’s Community Association	2014-11-05	2020-10-12
The Social Democrats (local political party)	2015-01-29	2020-09-23
Jokkmokk’s Snowmobile Association	2015-02-26	2020-10-22
Randijaur Village Association	2015-02-25	2020-10-18
Jokkmokk Iron Mines AB/ Beowulf Mining PLC (the mining company)	2015-12-09	2019-06-28

After the first interview, the representatives of four organizations (the Swedish Society for Nature Conservation, Destination Jokkmokk, the Social Democrats, and the company) either left or had relocated/been replaced, meaning that the second round of interviews were conducted with new representatives in these instances. The interviews followed a semi-structured format (Flick 2006) and included questions to capture both the diagnostic and the prescriptive components of the actors’ frames, including their perceptions regarding the planned mine, other involved actors, relationships, the regulatory framework, the policy/permit process, as well as their preferences towards the mine and advocated actions (van Hulst and Yanow 2016). Included in the second round of interviews were also questions to capture the reason for the actors’ reframing or frame stability. These focused on why the actors’ interview responses had changed or not between the two interviews. They included both open questions, to which the actors were able to freely give reasons for their changes/stability, and probing questions on the role of the various developments and events identified in the conflict.

All materials were analyzed in NVivo using qualitative thematic analysis (Spencer et al. 2014). The conflict was traced by analyzing documents (e.g., media reports, documents created by the actors, public documentation from the permit process, and regulatory documents) applying an open coding strategy. Specifically, all documents were carefully read and key developments and events relating to the micro-level (e.g., changes related to actors and their relations), process (e.g., intergroup communication and developments in the permit process), and macro-level (e.g., institutional changes) of the conflict were registered. The actors’ frames were traced based on the longitudinal interviews. This process involved, firstly, developing a thematic framework based on the diagnostic and prescriptive frame components (perceptions, preferences and advocated actions) presented above; familiarization with the data; and open test coding. Thereafter, the interviews were coded based on the framework and condensed into tables with summary statements corresponding to the themes. Tables with similar content were aggregated, enabling five distinct groups of frames to be generated, which were validated by the actors (see Table 2; see also Beland Lindahl et al. 2018 for a detailed description of the initial frames). The process was repeated for the two sets of interviews and the resulting frames were compared to reveal the actors’ reframing (see Supplementary Appendix A for a summary of the actors’ reframing). Important to emphasize is that the frames and reframing identified may not reflect all members in the respective organization. Nevertheless, as the actors were interviewed as organizational representatives, it is reasonable to assume that they reflect the organizations on a general level.

**Table 2:** Summary of the frames held by the actors in 2015

Frame groups	1. Reindeer husbandry consistent with nature	2. Local development consistent with nature	3. Multifaceted community and local benefits	4. More jobs, and growth	5. More mines and higher profits
Perceptions of issues	Landscape for reindeer herding with many values and functions is existentially threatened by the many negative effects of mining	Landscape and socioecological system with many values and functions is existentially threatened by the many negative effects of mining	Municipality with attractive living areas - yet in need of jobs and growth; mining might yield local benefits but must be assessed in relation to costs/risks	Municipality existentially threatened by unemployment and depopulation can be saved by the many positive effects of mining	Vast municipality with good conditions for mining that will yield many positive local, national, and international effects
Perceptions of actors and relations	Previously good community relations are under threat; some mine proponents are causing polarization	Previously good community relations are under threat; some mine proponents are causing polarization	Previously good community relations are under threat; actors with extreme positions are causing polarization	Previously good community relations are under threat; some mine opponents and the media are causing polarization	Community is in favor of mining; Opponents either are not from Jokkmokk or are Sami whose motives are land ownership
Perceptions of processes and institutions	Closed mining sector/permit process is dominated by the State and mining industry, and is in violation of Sami rights which should have priority	Closed mining sector/permit process is dominated by the State and mining industry and is in violation of Sami rights which should have priority	Mining sector/permit process is closed to local groups and interests which should have priority	Relatively fair and open mining sector/permit process; strong environmental legislation prevents negative effects	Relatively fair and open mining sector/permit process; strong environmental legislation prevents negative effects
Positions and actions advocated	Stop the mine, overhaul the legislation, strengthen Sami rights, and invest in small-scale local businesses (reindeer husbandry)	Stop the mine, overhaul the legislation, and invest in small-scale local businesses using renewable resources	Establish the mine if local benefits outweigh costs and damages; overhaul the legislation, and invest in local businesses for local benefits	Establish the mine and invest in the natural resource sector – mining can co-exist with other land uses	Establish the mine and a new mining district – mining can co-exist with other land uses

Finally, to specify the factors that enabled or inhibited reframing, transcripts from the second round of interviews were analyzed. Specifically, the answers to why the actors’ interview responses had changed (or not) and regarding the role of different developments and events were coded and analyzed to reveal the factors that mattered for the observed reframing or frame stability. The themes guiding the coding were developed based on the developments/events identified in the tracing of the conflict, as well as through an open, inductive coding of the interview transcripts, thus enabling previously undiscovered factors to be identified.

To give readers an understanding of how the empirical material has been interpreted, quotes (translated from Swedish) from, and references to, the data are included in the presentation of the results.

## Results

The results are presented in three sections. The first section gives a description of the conflict prior to, and the actors’ frames at the outset of, the study period. The second section presents developments during the conflict and the actors’ reframing during the study period. Finally, the third section presents the factors that enabled or inhibited reframing among the actors.

### The Conflict Prior to, and the Actors’ Frames at the Outset of, the Study Period

#### The conflict prior to the study period

The conflict was sparked when the company was granted an exploration permit from the Swedish Mining Inspectorate (MI) in 2006.^5^ As actors became aware of the project, division began to crystallize, and mobilization followed on different sides. In 2011, the resistance movement No Mines in Jokkmokk was launched (Tuorda et al. 2019), and the local political parties the Green Party and Sami Wellbeing declared that they were opposed to the mining operation (Lindh 2011). The year after, the nationwide mining skeptical movement Urbergsgruppen was formed, enabling the local mine opponents to build relations and protest across local and national levels. After the RHCs were informed about the project (2011) the two most affected RHCs, the Sirges and Jåhkågasska Sami, initiated collaboration (Jåhkågasska RHC and Sirges RHC 2014; Tuorda et al. 2019). Between 2011 and 2013 they launched several complaints and appeals against the company’s exploration activities, which were all dismissed (Mining Inspectorate 2011; Land and Environmental Court 2013).^6^ In 2013, the RHCs and No Mines in Jokkmokk informed UNESCO about the risk of a mine starting near the World Heritage Site Laponia, whereupon UNESCO started monitoring the case (Lundberg et al. 2013). Furthermore, using social media, local mine opponents managed to mobilize a wide range of mine critics from various parts of the country in protests and blockades, and as the police intervened with increased violence, national and international debates and media coverage were initiated (Moritz 2013). When the issue reached the national political debate, the Green Party at the national level declared support for the anti-mining position, highlighting the negative effects on the local Sami communities and Laponia (Isaksson 2013; Rosengren 2014). Support was also voiced by the Swedish Sami Parliament, highlighting the mine’s negative effects and its incompatibility with the Indigenous rights of the Sami (County Administrative Board Norrbotten 2013; Sami Parliament 2014).

Mobilizing the other side were actors largely in favor of mining. During the company’s exploration work, Randijaur Village Association rented its community building to the mining entrepreneurs, and villagers were promised employment in the mine (Interview, Randijaur Village Association 2015). Positive statements about the mine were also made by representatives of Jokkmokk Business Association, the Social Democrats in Jokkmokk, and Randijaur Village Association (Andersson 2013; Vikström 2011; Zerpe 2013). Moreover, a partnership was initiated between the company and the landowner association Jokkmokk Forest Common in 2014, which entailed a 500,000 SEK donation to the Forest Common for the purpose of supporting local business development (Sunna 2019). Several mine proponents also voiced criticism against the protests and blockades (Leijon 2013; Zerpe 2013), including the company whose series of controversial statements eventually led to replacement of its management in 2014 (Nordlund 2016). Although none of the national political parties declared their support for a mine in Kallak at this time, the mine proponents’ position was (and still is) indirectly supported by Sweden’s pro-mining politics/legislation, aimed to support the growth of the Swedish mining industry (Prop. 1998/89:92).

As the division within the community intensified (Sjöberg 2013), Jokkmokk municipality took action by organizing four citizens’ dialogues between 2013 and 2014. These dialogues included a broad range of local actors, the company, and the relevant agencies (Lind 2013). Concurrently, researchers involved in a project evaluating dialogue prospects between actors in NRM conflicts arranged three dialogue meetings (for a detailed description, see Johansson et al. 2022). Both the citizens’ dialogues and the dialogue meetings were designed to align with recommended principles outlined in previous research and theory on intergroup communication (see theory section).

In 2013, after 5 years of exploration, the company submitted an application for a mining permit, including an Environmental Impact Assessment (EIA), to the MI.^7^ Because of the halted dialogue between the RHCs and the company, the RHCs’ perspectives on the mine’s impact on reindeer herding were not reflected in the EIA (Hifab AB 2013).^8^ Consequently, the RHCs disputed the company’s EIA and developed their own assessment, which was submitted to the MI and the County Administrative Board (CAB)^9^ (Jåhkågasska RHC and Sirges RHC 2014). At the end of 2014, the CAB, as liaison authority for the EIA process, recommended, in line with the mine opponents’ position, that the permit should not be awarded because of the risk of irreversible negative effects on the land and existing land uses, including reindeer herding (County Administrative Board Norrbotten 2014).^10^ In response, the MI referred the matter to the Government, as is required when the assessments of the MI and the CAB differ. Specifically, the MI argued that the CAB had focused on aspects beyond the scope of the assessment and that it had not sufficiently weighed the land use interests within the specific mining concession area (Mining Inspectorate 2015).

#### The actors’ frames at the outset of the study period

Table 2 summarizes the frames held by the disputing actors in 2015, around the time when the company’s mining permit application was first assessed by the authorities (see Beland Lindahl et al. 2018 for a detailed description of the initial frames). The frames in groups 1 and 2 were shared by actors in clear opposition to the mine, i.e., the Sami RHCs Jåhkågasska and Sirges (group 1); No Mines in Jokkmokk (group 2); the local chapter of the Swedish Society for Nature Conservation (SSNC) (group 2); Björkholmen Village Association (group 2); and the local political party Sami Wellbeing (group 2). These frames shared many similarities, although reindeer herding and Sami rights were more prominent in the RHCs’ frames in group 1. The frames perceived the site of the planned mine to be part of a large, interconnected landscape with many values (ecological, recreational, cultural, and historical) and land uses that are not compatible with mining. Consequently, mining was perceived as a threat and was associated with many negative effects and risks. Community relations, likewise, were seen as threatened, particularly by the actions (e.g., exploration/application activities and public statements) of the company and some other mine proponents.

Prominent in the frames were also negative perceptions of the permit process and the institutional framework governing it (i.e., the permitting system). Specifically, the system was perceived as closed, dominated by the mining industry and a pro-mining State, and as being in violation of Sami rights. Consistent with these negative perceptions, the frames in groups 1 and 2 prescribed various decisions and actions to stop the project and change the legislation.

The frames in groups 4 and 5 were held by pro-mining actors, i.e., the Social Democrats, Jokkmokk’s Snowmobile Association, Randijaur Village Association (group 4), and the company (group 5). The frames in group 4 focused on what was perceived as existential threats to the municipality, from depopulation and unemployment. Accordingly, a mine was seen as an enabler bringing many positive local socioeconomic effects (e.g., jobs, growth, and increased/better services). The company’s frame (group 5) focused mainly on various mining-related conditions in the municipality (e.g., quality/size of the iron ore deposit, land area, and infrastructure), which were perceived as near perfect for mining. Moreover, the company’s frame associated mining with positive effects, not only locally, but nationally and in the context of the European Union (EU) as well.

Similar to the frames in groups 1 and 2, the local mine proponents’ frames perceived the community relations as threatened.^11^ Yet, these frames blamed polarization on the protests/blockades organized by the mine opponents, as well as the media coverage of the issues, which they perceived as biased. Present in the group 4- and 5-frames were also positive perceptions of the permitting system as a relatively open and fair system consistent with Sami rights, and with an environmental legislation that will prevent negative effects. Consistent with these positive perceptions the frames in groups 4 and 5 prescribed decisions and actions to realize mining.

Between the polarized frames (groups 1 and 2 vs. groups 4 and 5) were the frames in group 3. These articulated more cautious attitudes to mining and were identified among Jokkmokk Forest Common, Jokkmokk’s Community Association, and the business and rural development organizations Destination Jokkmokk (a local tourism organization), Jokkmokk Business Association, and Strukturum (an organization for local business development). The group 3-frames highlighted a municipality with attractive living conditions and recreational areas while also recognizing the problems of depopulation and decreasing services and employment opportunities. Accordingly, both positive (e.g., jobs, growth) and negative (e.g., environmental impact) effects of mining, as well as trade-offs, were included in the frames. Like the other local groups, the frames in group 3 perceived the community relations as threatened. They blamed polarization on actions from both sides in the conflict, particularly the blockades organized by some mine opponents and public statements made by the company and other mine proponents. The permitting system was also prominent in the frames in group 3. Specifically, the group 3-frames perceived the system as largely closed to local groups and interests, which, they believed, should have priority. In line with these perceptions the group 3-frames prescribed an overhaul of the legislation (to make it more attuned to local actors and interests) and certain conditions to granting a mining permit. These mainly concerned perceived environmental impact, local employment, the duration of operation, (Swedish) ownership, and local retention of profits.

### Developments in the Conflict and the Actors’ Frames during the Study Period

#### Developments in the conflict

The tracing of the conflict revealed a wide range of developments. Starting with the permit process, the company’s application was sent back and forth between the Government and the MI, with several rounds of comments from a wide range of actors, which demonstrated divided opinions. A key event that influenced the process following the 2014 national election was the formation of the coalition Government, consisting of the Social Democrats, a generally pro-mining party, and the Green Party, which expressed both skepticism towards mining (Green Party 2018) and opposition to the particular project (Rosengren 2015). The Government coalition lasted until 2021 and inter-coalition conflicts resulted in long periods of stalemate during which no visible measures were taken by Government (Committee on the Constitution 2020; Röstlund and Urisman Otto 2022). The first stalemate occurred at the beginning of 2015, after the MI referred the case to the Government following the disagreement between the MI and the CAB. This was broken in 2016, when a decision (concerning a different mine) by the Supreme Administrative Court (SAC) redefined the scope of the required EIA in terms of the Minerals Act (Supreme Administrative Court 2016 ref. 21). Specifically, the SAC ruled that an EIA must include, as well as the concession area, the mine’s adjoining activities and infrastructure.

Following this decision, the Government returned the application to the MI for a renewed assessment (Government 2016). The widened scope of the assessment meant that the impacts on the World Heritage Site Laponia were given a more prominent role, which led to new disagreements between the MI and the CAB concerning which authority had the mandate to assess these (Mining Inspectorate 2017). Consequently, the case was referred back to the Government. After the Government requested the CAB to make an assessment, the CAB recommended at the end of 2017, in line with its previous statement, that the mining permit should not be awarded because of the mine’s potential irreversible damage to surrounding land and land use, including reindeer herding, and the values of Laponia (County Administrative Board Norrbotten 2017). The CAB’s recommendation led to a second stalemate lasting until late 2020 (Röstlund and Urisman Otto 2022), when the Swedish Constitutional Committee initiated a review of the Government’s handling of the case. The Government turned to UNESCO for a statement on the mine’s impact on Laponia. The UNESCO statement, issued in 2021, concluded that the mine would impact Laponia, but that more information is needed for a complete assessment (UNESCO 2021).^12^

Parallel to this process, mobilization continued on both sides in the conflict. A general theme characterizing this mobilization was the increasing use of scales other than the local to get recognition and support. For example, the mine opponents increasingly invoked various national and international contexts and presented the mine as an example of state colonialism, an Indigenous/human rights violation, and/or a threat to Laponia (see, e.g., Dahlberg and Fröberg 2018; Borg 2020; Stokki 2020). This generated publicity in new settings, and support was gathered from a wide range of actors, many of whom had previously been silent on the issue. They included non-governmental organizations (NGOs) (such as Greenpeace and Amnesty), green movements and activist networks (e.g., Fridays for Future, Rainbow Gatherings, and the International Gállok Protection Camp), and international bodies such as UNESCO and the Committee on the Elimination of Racial Discrimination (CERD) (Olofsson 2018; Wänkkö and Stridsman 2019; Poggats 2020; Jakobsson 2020). In addition, by maintaining the support of the Green Party at the national level, the mine opponents were able to advance/safeguard their position in the formal permit process during the Government’s handling of the issues (Röstlund and Urisman Otto 2022). This was evident on several occasions when leading figures in the party declared that they are doing what they can to stop the mine and strengthen Sami rights (Rosengren 2015; Dahlberg and Fröberg 2018).

Mobilization among the mine proponents exhibited similar signs of scale-jumping. Locally, the new company management made several visits to Jokkmokk to meet with actors and build relationships and support (Beowulf Mining 2018; 2019). During these, plans and agreements were developed (e.g., with the Social Democrats and Jokkmokk Forest Common) to create local readiness in case of the project’s approval (Beowulf Mining 2018; Vikström 2019). An additional donation of 300,000 SEK to the Forest Common was also promised (Sunna 2019). Attempts to reach agreement with the affected RHCs were also made, but the RHCs stated that they see little value in engaging with the company (Skoglund 2015). Nationally and internationally, pro-mining mobilization consisted of various actions in national media, Organization for Economic Cooperation and Development (OECD) forums, and the Politician’s Week in Almedalen^13^ (see, e.g., Beowulf Mining 2018; 2019; Vikström 2019). In these contexts, Sweden’s decline in the Fraser Institute’s Annual Mining Survey,^14^ the increasing proportion of Swedish shareholders in the company, and the mine’s contribution to Sweden’s post-pandemic recovery and transition to green energy/technologies were highlighted (Beowulf Mining 2020; Höglund 2020; Nilsson and Forsberg 2020).

During the study period, several actors voiced increasing support for the mining operation, including a wide range of local/regional entrepreneurs and business organizations, and politicians who had previously been silent on the issue (Kvickström et al. 2017; Stenberg et al. 2018). National support included indirect support through Sweden’s pro-mining politics/legislation, and direct support from the Social Democrats, who advanced the mine proponents’ position in the formal permit process (Röstlund and Urisman Otto 2022). The support from the Social Democrats became particularly evident when the Green Party left Government and the Social Democrats declared, in line with the State’s general pro-mining position, that they “love mines” and promptly granted the company a mining permit (Liikamaa 2022).

In addition to the developments in the permit process and the actors’ mobilization, several intergroup communicative processes were hosted. As a part of the new company management’s strategy to build/repair relationships, a dialogue between the affected RHCs and the company was held in 2015 (Skoglund 2015), and open information meetings were held in Jokkmokk between 2017 and 2020 (Beowulf Mining 2018; 2019; 2020). Moreover, as part of a research project exploring the prospect of dialogue in the conflict, two dialogue meetings were organized in Jokkmokk in 2015 (June and November) in accordance with the prescribed design principles outlined in the theory section (see Johansson et al. 2022 for a detailed description of the meetings).

Finally, a number of institutional developments during the study period included the previously mentioned SAC ruling in 2016 concerning the widened scope of the EIA, which prompted the Government to return the application to the MI for a renewed assessment (including the impacts on Laponia) (Supreme Administrative Court 2016 ref. 21). Furthermore, to improve public participation and the prioritization between different land use interests, as well as reduce the risk of appeals, consultations relating to EIAs in the mining permit application process were made mandatory in 2018 (Prop. 2016/17:200). However, since this change came after the company had submitted its EIA, it had little bearing on the permit process. Apart from these developments, the overarching goal of Swedish mining politics and the permitting system remained the same, i.e., to facilitate exploration activities and mining developments to support the growth of the Swedish mining industry (Prop. 1998/89:92).

Against the backdrop of key developments in the conflict, the next section presents the reframing that was identified over the course of these (see Supplementary Appendix A for a complete summary of the actors’ reframing).

#### The actors’ reframing

The frames in groups 1 and 2 largely remained the same. These frames continued to perceive the mine as an existential threat and the permitting system as closed, dominated by pro-mining interests, and in violation of Sami rights. Accordingly, they also continued to advocate decisions and actions to stop the mine and change the legislation. Nevertheless, several changes were identified, many of which contributed to bringing the two groups closer together. For example, increased focus on the conditions for/effects on reindeer herding, as well as a stronger emphasis on strengthened Sami rights was noted in the frames in group 2:

The core problem is that the mine will have a profound impact on reindeer husbandry … If the reindeer herders express their disapproval, it should not be possible to approve the project. (Interview, group 2 2020)

Furthermore, in all frames in groups 1 and 2, the mine project was more clearly represented as a violation of Sami/Indigenous rights, and a critical point in State–Sami relations in 2021, compared to 2015, in which local conditions and effects was in focus. Moreover, a significant increase in expression of solidarity and cooperation between the mine opponents was identified in the frames in group 1 and 2:

Our collaboration has improved a lot since the last time … it is truly amazing to witness this growing solidarity, not only between us here in Jokkmokk but also internationally. (Interview, group 1 2020)

In addition, with a few exceptions (see below), the group 1- and 2-frames developed more positive perceptions of the community and intergroup relations. Specifically, previous descriptions of community relations as being threatened by the mine proponents’ polarizing actions had largely been replaced with descriptions of a pleasant community with respectful relationships:

The atmosphere in Jokkmokk is pleasant today. We no longer have to fight because we have a clear understanding of our positions. Of course, there will always be a few malicious individuals … but overall, people are respectful. (Interview, group 2 2020)

However, the frame of the SSNC diverged from this pattern, showing more negative perceptions of the relations between different actors in the community (increased division). Furthermore, more negative descriptions (using terms such as “problematic” and “threats”) of the company and the municipal leadership (primarily the Social Democrats) and their efforts to mobilize opinion in favor of the mine were noted in all frames in groups 1 and 2:

We have a company that possesses significantly greater expertise, coupled with a municipal leadership that wants to push forward with the project regardless of the consequences … This presents greater challenges for us. (Interview, group 1 2020)

Apart from the frame of Randijaur Village Association (see below), the frames of the actors strongly in favor of the mine (groups 4 and 5) also remained largely unchanged. Hence, the frames continued to perceive the mine as an enabler with many positive effects, and advocate decisions/actions to establish the mine. The frames also, like the mine opponents’ frames, converged during the study period. Specifically, the local mine proponents’ frames in group 4 incorporated several aspects of the company’s frame (e.g., emphasis on national and international effects of mining), while the company’s frame incorporated many aspects of the local mine proponents’ frames (e.g., emphasis on local conditions/benefits). Improved intragroup relationships and cooperation between mine proponents were also identified in the frames:

Today, the company’s focus lies in becoming a local partner instead of operating independently … This has resulted in a positive collaboration taking place here in Jokkmokk. (Interview, group 4 2020)

However, the frame of Randijaur Village Association diverged from these patterns in that its initially pro-mining perceptions/position changed to indecision (i.e., mining if local benefits outweigh costs and damages), as expressed in the frames in group 3. In addition, this frame developed a negative perception of the company, from being an actor with a high local presence, wanting to benefit the community, to being an absent actor with unclear motives:

We are considerably more skeptical about the mine today… For us to take a positive stance, it must first be ensured that there will be proper compensation for stakeholders and the municipality … nothing with the new management indicates that this will be the case … we haven’t seen them here in Jokkmokk. (Interview, group 4 2020)

Like most frames in groups 1 and 2, the group 4-frames’ perceptions of the community and intergroup relations became generally more positive (respectful/pleasant community and relationships):

The conflict is still present, but it is definitely not as infected as before … today we can talk to each other without fearing personal attacks. (Interview, group 4 2020).

However, the Social Democrats’ frame diverged from this pattern. Similar to SSNC’s frame, it developed more negative perceptions of the relations between different actors in the community (increased division).

Noted in the group 4- and 5-frames were also significantly more negative perceptions of the permitting system. From perceiving the system as relatively open and fair in 2015, they came to perceive it as largely flawed in 2021. The highlighted “flaws” concern the processing of issues, which was regarded as unacceptably slow; the authorities’ statements/assessments and the legislative provisions guiding these, which were perceived as arbitrary and unclear; and the Government’s inability to reach an agreement/decision. Accordingly, an overhaul of the legislation came to be added to the frames in group 4 and 5:

It is embarrassing that the system is so unclear that the authorities cannot make decisions… something needs to be done about it. (Interview, group 4 2020).

Midway between the polarized frames at the outset of the study period were the frames in group 3. Among these frames, more substantial changes were identified. However, the content of these frames developed in largely opposite directions, thus polarizing the group. Specifically, the frames of Jokkmokk’s Community Association and Destination Jokkmokk developed more negative perceptions of, and positions towards, the mine (the mine as a threat to an attractive municipality; stop the mine); more negative perceptions of the company (locally absent actor with unclear motives); while they maintained their previous perceptions of the permitting system (closed for local interests which should have priority). Hence, the frames developed many similarities to the mine opponents’ frames in group 1 and 2.

The frames of Strukturum, Jokkmokk Forest Common, and Jokkmokk Business Association, on the other hand, developed many similarities to the mine proponents frames in group 4 and 5. Specifically they developed more positive perceptions of, and positions towards, the mine (the mine as an enabler/asset in a struggling municipality; establish the mine); more positive perceptions of the company (a local benefactor and partner); and more negative perceptions of the permitting system (slow process and arbitrary/unclear assessments/rules). Despite this polarization, the frames in group 3 showed, similar to the other frame groups, generally more positive perceptions of the community and intergroup relations (“pleasant” and “respectful”). However, the frame of Jokkmokk Forest Common, diverged from this pattern, developing more negative perceptions of community relations (increased division), not unlike the frames held by the SSNC and Social Democrats.

### Factors that Enabled or Inhibited Reframing

Through interviews and interpretive work, four factors that mattered for the actors’ reframing or frame stability were identified: the nature of the issue, the actors’ intragroup relationships, intergroup communication, and the permitting system.

#### The nature of the issue

The first factor concerns the nature of the mine project. In interviews with mine opponents with frames in groups 1 and 2, in which a key priority/concern is the protection of the landscape, the maintained/stable negative perceptions of, and positions towards, the project were consistently connected to the inevitability of the impact of mining on the landscape and its incompatibility with the current land uses. According to the actors, these impacts made it difficult to regard the project as anything but a threat:

The mine will cut off our grazing lands … there are simply no compromises to be found with a mine … because reindeer cannot eat money … this is about our survival. (Interview, group 1 2020)

On the other hand, in the interviews with mine proponents and initially indecisive actors with frames in groups 3–5, in which the socioeconomic development in the municipality is a key concern, the reframing of, or stability in, perceptions/positions of/towards the project was consistently linked to socioeconomic benefits associated with the mine. Initially indecisive actors who developed more positive perceptions/positions (Strukturum, Jokkmokk Forest Common, and Jokkmokk Business Association) described how their initial uncertainty and questions regarding how the project would benefit the community had been addressed by the company, thus enabling a more positive view:

The company has shown the intention to do good here in Jokkmokk … of course that affects our position. (Interview, group 3 2020)

In a similar vein, actors who developed more negative perceptions/positions (Jokkmokk’s Community Association, Destination Jokkmokk, and Randijaur Village Association) described how uncertainties regarding the project’s local benefits had been generated during the study period, leading to a more negative stance:

The company once had the ambition to run the mine as a local project … now there is a large risk that it’s just going to be fly-in-fly-out … the money won’t stay here … nobody wants that. (Interview, group 3 2020)

Lastly, the mine proponents who maintained positive perceptions/positions (the Social Democrats, Jokkmokk’s Snowmobile Association, and the company) described that the mine’s many positive socioeconomic effects made it difficult to regard the project as anything but an asset for the municipality:

The mine is an asset for Jokkmokk … there is no option not to proceed with the project … Jokkmokk needs considerable economic activity and jobs to support municipal services … the mine can provide that. (Interview, group 5 2019)

Hence, the actors linked their reframing, or frame stability, to different aspects of the project. Although difficult to specify as a factor external to the actors’ frames, it is evident that a mine provides actors with different opportunities for reframing depending on its degree of compatibility with the different frame-groups (including the place-based practices and organizations which upholds them). As indicated by the findings, regardless of compensatory measures and intergroup communication, the inevitable impacts of an open-pit mine on the landscape and its land uses were not possible to reconcile with the group 1 and 2-frames’ key priority to protect the landscape, which, in turn, inhibited positive perceptions of, and positions towards, the project. On the other hand, in the frames in groups 3–5, in which socio-economic benefits are a key priority that can outweigh/compensate the inevitable impacts of mining, a mine offers more flexibility, and both positive and negative perceptions/positions were accordingly developed depending on the perceived benefits.

#### The actors’ intragroup relationships

The second factor identified concerns the actors’ intragroup relationships. Actors on both sides in the conflict emphasized the significance of their collaborations and relationships in contributing to the convergence of frames within their respective factions. According to the mine opponents, continuous protest activities and intragroup engagement in e.g., Facebook-groups enabled a strong sense of solidarity, sustained communication and, through this communication, the development of shared perceptions and preferences in the conflict:

We have had great collaborations with the RHCs, and with organizations … and Facebook has been great for building and exchanging knowledge and information about the project … This has also enabled us to develop a common understanding and strategy. (Interview, group 2 2020)

Likewise, local mine proponents and initially indecisive actors who developed increasingly similar frames in favor of the mine (groups 3 and 4) emphasized the significance of their enhanced relationships and sustained engagement with the company in driving these developments:

Our collaboration is no secret … it means that all information about the mine is given to us directly by the company … this has given us a common understanding and, to some extent, acceptance. (Interview, group 3 2020)

Hence, the strengthening of relationships and heightened engagement between actors who shared compatible frames played a crucial role in facilitating a greater convergence of frames on both sides of the conflict’s dividing line.

Evident in interviews was also that actors’ intragroup relationships worked against reframing that were incompatible with, or threatening to, these relationships. Specifically, several mine opponents described that their resistance was driven by a sense of solidarity for, and loyalty to, the broader environmental issues and/or Indigenous struggle of which they had increasingly become a part, and that this made it difficult to consider an alternative position in the conflict:

The issue has grown, and resisting this project has become so fundamentally important, not only for our survival, but for Indigenous peoples everywhere … so we can never accept this project … even if we’d like to. (Interview, group 1 2020)

#### Intergroup communication

The third factor concerns the intergroup communicative processes hosted during the conflict. However, not all processes were regarded similarly. Two processes that were often grouped together by the actors were the researcher-led dialogue meetings and the municipality’s citizens’ dialogues hosted outside of the framework of the formal permitting system. A common narrative was found across the actors’ descriptions of these processes. Although criticized by some for not being tied to formal decision making, the processes were generally described as neutral, inclusive, equal, and deliberative:

I was really surprised with how well the meetings turned out … people from all sides were there … it [the discussion] was heated in the beginning, but the mediator ensured good discussions where everyone got to speak their mind without interruptions. (Interview, group 2 2020)

Furthermore, all actors who developed more positive perceptions of the relations between the different actors in the community linked these changes to either one or both processes, describing how the processes shifted the community discourse away from hostile and aggressive discussions (including personal attacks) to more friendly and respectful dialogue:

By providing a public arena for discussion, the aggressive and hostile atmosphere changed … of course there are still heated discussions and provocations … but most of us have realized that we can actually talk about these things without hating each other … so yes, they have been very valuable. (Interview, group 3 2020)

When probed about how these developments/shifts had occurred, the general response was that the reciprocal sharing of opposing perspectives developed a better understanding of different actors’ frames and concerns, which in turn spilled over into a higher level of mutual respect:

The more we met the better it got … people actually started listening to each other … it made us understand each other’s perspectives much better … it created a form of respect because we realized that we all have good intentions. (Interview, group 4 2020)

The actors that developed more negative perceptions of community relations (the SSNC, Jokkmokk Forest Common, and the Social Democrats) also regarded the researcher-led dialogue meetings and the citizens’ dialogues as important for de-escalating the conflict, as well as viewing them mostly as high-quality processes that enabled a better understanding of different frames and a higher level of mutual respect. Yet, according to them, the positive impacts these processes had on community relations were outweighed by continued mobilization by different actors.

Hence, the intergroup communicative processes that were regarded as neutral, inclusive, equal, and deliberative enabled the actors to develop generally more positive perceptions of the community relations, a better understanding of each other’s frames, and a higher level of mutual respect.

Other processes highlighted by the actors were the company’s information meetings, as well as the corporate consultations which, in contrast to the dialogue meetings and citizens’ dialogues, were hosted within the framework of the formal permitting system. These were also grouped together in the actors’ descriptions. However, with regard to these processes, the different actors were highly divided. Among the mine proponents and those who were initially indecisive (groups 3–5), the processes were described as relatively fair processes that ensured that most perspectives and concerns about the project could be raised and registered, thus enabling a safe assessment of the project. Yet, a more inclusive range of participants was requested by some actors, as were more processes in the early (exploration) stages of the permit process:

These processes should be initiated earlier … but I think the company has worked methodically and talked to as many people as possible to ensure that all aspects of the project are covered. I know they have some issues with the RHCs, but I think they can be solved. (Interview, group 3 2020)

The mine opponents in group 1 and 2, on the other hand, shared extremely negative descriptions of the company-led processes, particularly the corporate consultations. Specifically, four related narratives reoccurred: (1) the processes are geared towards narrow and predetermined agendas and outcomes (i.e., to realize mining); (2) limited rights (narrow definition of rights/stakeholders) and resources (e.g., time and money) are provided for inclusive and equal participation; (3) participation in the processes is limited to the sharing and gathering of information about the mine’s impacts based on the premise that mining is inevitable and co-existence (between competing land uses) is possible, which consequently excludes the option of no mine being seriously considered and realized; and (4) the processes do not respect Sami Indigenous rights (e.g., the State has a duty to consult with the Sami prior to decision making, and Sami consent is required for encroachments on their traditional land areas).

Hence, in contrast to the researcher-led dialogue meetings and the citizens’ dialogues, the corporate consultations and the company’s information meeting did not enable reciprocal intergroup communication that were regarded by all actors as neutral, inclusive, equal, and deliberative. Moreover, there were no indications in the interviews that the company-led processes had any positive impacts on the intergroup relations in the community.

#### The permitting system

The fourth factor identified concerns the mine permitting system. Among the mine opponents in group 1 and 2, the permitting system was consistently described as an obstacle to meaningful intergroup communication and reframing. Specifically, the actors described the system as unjust, lacking opportunities for equitable participation/influence in decision making. Accordingly, several actors stated that they found protests more meaningful for achieving their goals than engaging/communicating with the company within in the framework of the permitting system (e.g., in corporate consultations):

We have hardly participated in consultations because there is nothing to consult about. Protests are what we can use to achieve change …. If we could influence the permit process, it would be more interesting to sit down and talk to the company. (Interview, group 1 2020)

Moreover, the RHCs often described the meetings with the company as a necessary evil they were forced to participate in:

It’s like talking to burglars before they commit a break-in …. The question is not if they will take your stuff, but rather, when, and how. But still, we need to talk to them to ensure that they don’t take everything. (Interview, group 1 2020)

Hence, because the mine opponents perceived the opportunities they had to advance their objectives within the permitting system as limited, they lacked incentives to engage with their disputants within the system. As a result, meaningful intergroup engagement/communication between the disputing actors were not realized within the framework of the permitting system.

As presented above, most frames in groups 3–5 developed significantly more negative perceptions of the mine permitting system during the study period. Accordingly, when the topic of the permitting system was brought up in the interviews with the actors sharing these frames, the primary focus was to identify the factors that enabled these developments. In the interviews, the actors linked their changed perceptions of the system to three aspects of the permit process: (1) the long time span of the process, which they regarded as unacceptable, and as an obvious sign of a flawed system; (2) the assessments/statements (concerning the priority of different land uses) made by the authorities in the process: To them, the authorities’ conflicting assessments and statements made it evident that the system suffered from a large degree of arbitrariness and unclarity; and (3) the Government’s handling of the issues. The general understanding was that the Government parties (the Green Party and the Social Democrats) deliberately delayed processing the issues to avoid controversy and criticism from their constituencies, thus failing to fulfill their duty:

This whole process has been a disaster. It has been slow and unclear. It feels like the authorities can interpret the law however they want …. This shows that the system is not secure - frankly, it’s a failure. The Government’s treatment of the case is also reprehensible …. If you’re afraid to make tough decisions, you shouldn’t rule. (Interview, group 3 2020)

As many developments in the permit process (e.g., the assessment by the CAB, the ruling by the SAC, support from the Green Party, and the involvement of UNESCO) seemed well-aligned with the mine opponents’ frames (groups 1-2), it would be logical to expect more positive perceptions of the permitting system among these actors. Yet, as shown above, the mine opponents maintained their negative perceptions of the system. When probed about this stability, the actors said that more positive perceptions of the system would require more radical, and permanent, changes that ensure that different values (ecological, social, cultural, and traditional) and land uses (including the livelihood of the Indigenous Sami) are protected in the legislation, and do not dependent on uncertain aspects such as: the ideological leaning of the Government; the discretion of an individual authority; the resources of an individual RHC; or company goodwill. The changes suggested included greater opportunities for inclusive and equal participation and influence; the recognition of Sami consent for encroachments on Sami land areas; and reforms that make the legislation more attuned to values and land uses other than commercial.

## Discussion

The present study has explored to what extent, how, and under what conditions reframing can occur in intractable NRM conflicts.

### The Extent to Which, and How, Reframing can Occur

In line with frame theory and previous research (Perri 6 2005; Putnam 2010; Rein and Schön 1996), the study found that reframing is difficult to enable in intractable conflicts. Despite many attempts at dispute resolution, including high-quality intergroup communication, the conflicting frames, with clear positions for or against the mine, largely remained stable throughout the 5-year study period. Moreover, no signs of consensus-oriented reframing were identified. On the contrary, most reframing that occurred contributed to increased polarization: First, the frames on each side of the dividing line of the conflict converged, making the divide between the already polarized frames more pronounced. Second, the frames positioned midway between the outer frames at the outset of the study period polarized, thus furthering the division. Hence, the study supports the argument that consensus is an improbable outcome in intractable conflicts (Johansson et al. 2022; Kahan et al. 2011; Terpstra et al. 2009).

Some scholars advocate meta-consensus as a more desirable and realistic outcome in conflict (Curato et al. 2017; Dryzek and Niemeyer 2006). Meta-consensus applies to a situation where the present frames are mutually understood and accepted by all actors. Several of the study’s findings indicate that meta-consensus was enabled through reciprocal intergroup communication (see discussion in the section below). For example, in all groups, the initial description of the intergroup relations in the community as being threatened by polarizing actions and an increasingly hostile climate was largely (but not entirely) replaced by descriptions of a pleasant community/discourse with respectful relationships between disputants. Moreover, in the interviews, actors across all groups confirmed that they had developed a better understanding of each other’s frames and, as a result, a higher level of mutual respect. Hence, the study largely supports the argument that meta-consensus can be enabled in intractable conflicts (Curato et al. 2017; Johansson et al. 2022; Niemeyer 2013). Meta-consensus is a valuable outcome that turns conflict from a struggle to eradicate each other’s frames, to disagreement in which all frames can be respected and heard. It is also argued that, by clarifying the conflict and its divisions, meta-consensus provides a ground for tractable decisions (Dryzek and Niemeyer 2006). However, additional research is necessary to determine whether and how meta-consensus can be effectively translated into legitimate decisions. As indicated in this study, the current Swedish mine permitting system does not possess procedures and mechanisms to accomplish this objective.

### Conditions that Enable or Inhibit Reframing

In line with previous research and theory (Buchecker et al. 2021; Putnam 2010; Rein and Schön 1996), the study found that intergroup communication based on recommended design principles can enable mutual frame reflection and reframing. However, as discussed above, consensus did not occur, but the intergroup communicative processes that were aligned with the recommended design principles (i.e., the researcher-led dialogue meetings and the municipality’s citizens’ dialogues) enabled the actors to develop a better understanding of each other’s frames and higher levels of mutual respect, i.e., reach meta-consensus. This finding is in line with recent deliberative research showing that meta-consensus can be enabled in intractable conflicts when the disputing actors come together under conditions of equality and fairness to reciprocally discuss issues and listen to the full range of competing frames (Curato et al. 2017; Johansson et al. 2022; Kuyper 2018). However, nothing in the results suggests that the communicative processes initiated within the framework of the formal governance/permitting system (i.e., the corporate consultations) contributed to meta-consensus. On the contrary, the results indicate that these processes were unable to facilitate neutral, inclusive, equal, and deliberative intergroup communication. This can be attributed to several factors, such as the narrow selection of participants, the absence of adequate compensation for participation, and the narrow agendas and objectives focused solely on assessing, sharing, or gathering information about the mine’s impacts on a limited scale. These findings underscore the importance of understanding intergroup communication as embedded in governance/institutional contexts which shape both processes and outcomes in powerful ways (Arai et al. 2021; Baker and Chapin III 2018; Raitio 2013). Considering this finding, future efforts should focus on how governance systems, such as the Swedish mine permitting system, can be effectively addressed to foster fruitful intergroup communication in formal decision making processes.

Furthermore, the study revealed that the nature of the issue under dispute influences the potential for reframing in intractable NRM conflicts. Specifically, the inevitable impacts of the planed mine project clashed with the core priority of the mine opponents’ frames, which aimed to protect the landscape and existing land from (further) encroachments. This led to the development of unwavering negative perceptions and positions among actors holding these frames. Conversely, for the indecisive actors and mine proponents, whose frames emphasized the socioeconomic benefits of mining, the project offered more flexibility. Consequently, both positive and negative perceptions and positions were developed among these actors based on the perceived socioeconomic advantages of the project. These findings underscore the importance of recognizing disputed issues not solely as aspects of frames, but also as external factors which offer varying opportunities for reframing among actors in conflict. It is widely acknowledged that mining causes more damage to the natural environment compared to many other industrial sectors (Conde 2017). Therefore, expecting actors whose livelihood largely depends on utilizing the land on which mining is planned to openly embrace a positive stance towards the planned project might not be realistic.

Additionally, the study found that actors’ commitments and loyalties to intragroup relationships influence the potential for reframing in intractable NRM conflicts. Specifically, the forging and strengthening of intragroup relationships enabled more similar frames on each side of the main dividing line of the conflict, while also inhibiting reframing that was seen as incompatible with these relationships or as a threat to them. These findings are in line with frame theory which highlights actors’ commitments to social norms within their network of relationships (i.e., their organizations) as important for how they perceive, and act in, the world (Schön and Rein 1994; Perri 6 2005). These norms help actors stay connected, maintain a sense of community, and act collectively, but they also constitute hurdles for reframing. Hence, grasping the prospects for reframing in a conflict requires that the actors’ social relationships are considered, as there are limits to what types of reframing that are possible for actors who want to uphold these (Perri 6 2005). For instance, it is highly improbable that mine opponents who are deeply committed to environmental and Indigenous rights movements would intentionally act and reframe their perspectives in a manner that supports the advancement of the mine.

Finally, in line with previous research highlighting how power imbalances can discourage actors from engaging in communication in intractable conflicts (e.g., Arai et al. 2021; Baker and Chapin III 2018; Raitio 2013), the study found that the mine permitting system inhibited reframing by failing to provide sufficient incentives for the mine opponents to engage with their disputants. Specifically, because the mine opponents felt that they had few or no opportunities to advance their positions in the formal system, many considered informal actions and intragroup mobilization as more meaningful than engaging with their disputants in the permit process (e.g., through participating in corporate consultation). Accordingly, although some few opportunities existed for disputing actors to communicate across group lines in the system, the mine opponents often chose not to communicate as they saw little value in doing so. This phenomenon can be explained with the concept of the “Best Alternative to Negotiated Agreement” (BATNA), or the idea that parties do not negotiate or even communicate if they believe that they can advance their positions better without doing so (Pölönen et al. 2020; Susskind et al. 1999). Addressing this situation requires contextual or institutional changes that level power imbalances to the extent that all disputants perceive intergroup engagement as a better option than unilateral actions. In the case of Swedish mine governance, significant reform efforts are needed to ensure that all actors have meaningful opportunities to participate in, and influence, decision making. Moreover, if Sami Indigenous rights are not integrated and strengthened in the system, it is unlikely that the mine opponents will regard the system as legitimate.

## Conclusions

Increasing competition for the world’s natural resources will likely escalate and aggravate intractable conflicts. Regardless of the outcome deemed most desirable, in managing these conflicts it is beneficial to understand to what extent, how, and under what conditions reframing is possible. The study can conclude that the prospect of achieving reframing towards consensus, or a mutually accepted agreement, is bleak. Not only were no signs of consensus-oriented reframing identified, but the actors’ frames became increasingly polarized during the conflict, despite high-quality intergroup communication. This is an important finding that needs to be considered in NRM practice which increasingly relies on communicative efforts and consensus-oriented reframing to manage escalating conflicts.

Nevertheless, the study suggests that intergroup communication, when organized according to recommended design principles, can effectively enable reframing to the extent that all conflicting frames become mutually understood and accepted, leading to the achievement of meta-consensus. Meta-consensus is a valuable outcome in that it transforms conflict into a structured disagreement where traditionally marginalized actors and frames are respected and acknowledged. Meta-consensus also facilitates the search for agreements when they are attainable and fair choices or trade-offs when they are not. This underscores the importance of properly designed and facilitated communicative processes in intractable conflicts. However, it is important to recognize that meta-consensus alone does not guarantee a legitimate decision or agreement. Complementary efforts should therefore be directed towards identifying legitimate mechanisms for making acceptable trade-offs and choices in the light of meta-consensus. Furthermore, considering that meaningful intergroup engagement and meta-consensus was not achieved within the framework of the formal permitting system in the investigated case, it is vital to prioritize efforts to identify how governance systems can be optimally configured to facilitate fruitful intergroup communication and reframing in formal decision making processes.

Finally, the study can conclude that reframing in conflict is influenced by a wide range of institutional and contextual factors, beyond the quality of intergroup communication. These factors include the nature of the disputed issues, actors’ commitments and loyalties to intragroup relationships, and the distribution of power within the governance system embedding the conflict. The present study has only begun to explore the intricate interactions and influences of these factors on reframing. To further advance the knowledge on what types of reframing that can be enabled under different conditions, more research on the interrelationship between context, intergroup communication, and reframing is needed. As demonstrated in this study, frame analysis and interpretive process tracing offer practical tools for advancing this type of knowledge.

## Supplementary information

Supplementary Information

## Footnotes

1 Laponia has been granted World Heritage status for its outstanding nature conservation interests and unique Sami culture.

2 Swedish Sami reindeer herding is divided into 51 reindeer herding communities (RHCs), which are economic associations as well as geographical areas. Members of RHCs own special rights to reindeer husbandry, which includes reindeer herding, hunting and fishing. The planned mining site is located on land situated on Jåhkågasska RHC reindeer grazing and migration routes; the planned transportation routes will cross the Sirges RHC’s grazing areas.

3 The Minerals Act is the principal law regulating the mining industry and the procedure for acquiring exploration and exploitation permits.

4 Following an inductive logic, the study was conducted over a long period to ensure that the conflict, and the conditions of the conflict, had plenty of time to develop and change.

5 The Mining Inspectorate (MI) is responsible for permitting and supervision under the Minerals Act. An exploration permit gives access to the land and an exclusive right to explore within the permit area. It is the first of four steps in the Swedish mineral extraction permitting process, and is followed by a mining permit, an environmental permit, and land designation.

6 The appeals were submitted by Jåhkågasska because only property owners and special rights holders (e.g., the RHCs) immediately affected by exploration work or the mining concession are formally entitled to submit statements and appeals, according to the Minerals Act.

7 A mining permit gives the holder the right to exploit a proven extractable mineral deposit for a period of 25 years, which may be prolonged.

8 Until 2018, there was no legal requirement for a mining company to consult any local actors in the mining permit application process. Therefore, the halting dialogue did not constitute a legal obstacle.

9 The County Administrative Board (CAB) has responsibility for permitting and supervision under the Environmental Code.

10 The mining permit decision is preceded by an assessment of which opposing public/national interests (land uses) at the location of the proposed mine should be given precedence. In the assessment, the Mining Inspectorate (MI) consults the CAB which coordinates and safeguards the public/national resource management (NRM) interests in the region.

11 The company was mainly silent on elements concerning other actors and relations. Yet their frame described a community in favor of mining, stating that mining opponents were either outsiders (i.e., not from Jokkmokk) or Sami whose motives were to gain land ownership.

12 After the study period, in November 2021, the Green Party, dissatisfied with the new approved Parliamentary budget, left the Government. In March 2022, the new Social Democratic Government granted the company a mining permit.

13 The Politician’s Week in Almedalen is an important forum in Swedish national politics that takes place annually in Visby.

14 The survey is an attempt to assess how mineral endowments and public policy factors such as taxation and regulatory uncertainty affect exploration investment.
