# Supplementary material for: Managing Intractable Natural Resource Conflicts: Exploring Possibilities and Conditions for Reframing in a Mine Establishment Conflict in Northern Sweden
Source: Environ Manage. 2023 Jun 8;72(4):818–37. doi: 10.1007/s00267-023-01838-5 (PMC10460354; doi:10.1007/s00267-023-01838-5)
Supplement: Supplementary file 1 — Supplementary Information [file 267_2023_1838_MOESM1_ESM.docx]

**Appendix A. Summary of the actors’ reframing between 2015-2021**

| Frame groups | 1. Reindeer husbandry consistent with nature | 2. Local development consistent with nature | 3. Multi-faceted community and local beneﬁts | 4. More jobs, and growth | 5. More mines and higher profits |
| --- | --- | --- | --- | --- | --- |
| Actors | A: Jåhkågasska reindeer herding communiity  B: Sirges reindeer herding community | C: No Mines in Jokkmokk  D: Swedish Society for Nature Conservation  E: Björkholmen Village Association  F: Sami Wellbeing | G: Strukturum  H: Jokkmokk Forest Common  I: Jokkmokk Business Association  J: Destination Jokkmokk  K: Jokkmokk’s Community Association | L: The Social Democrats  M: Jokkmokk’s Snowmobile Association  N: Randijaur Village Association | O: The mining company |
| Changes in perceptions of issues | A & B: Previous perceptions maintained, but views of mining as colonialism and a Sami rights violation are more prominent | C, D, E, & F: Previous perceptions maintained, but negative effects on reindeer herding, and views of mining as colonialism and a Sami rights violation are more prominent | G, H, & I: A more positive perception of mining: an asset for a struggling municipality  J & K: A more negative perception of mining: a threat to an attractive municipality | L & M: Previous perceptions maintained, but national effects of mining are more prominent: contribute to the green transition  N: A more negative perception of mining: benefits as well as costs/risks | O: Previous perceptions maintained, but local effects of mining are more prominent: an asset for a struggling municipality |
| Changes in perceptions of actors and relations | A & B: A more positive perception of the community and intergroup relations; respectful tone used by all but a few mine proponents  A & B: Perception of greater solidarity among mine opponents  A & B: A more negative perception of the company and the municipal leadership: a bigger threat | C, E, & F: A more positive perception of the community and intergroup relations; respectful tone used by all but a few mine proponents  D: A more negative perception of the community and intergroup relations: some mine proponents continue to cause polarization  C, D, E, & F: Perception of greater solidarity among mine opponents  C, D, E, & F: A more negative perception of the company and the municipal leadership: a bigger threat | G, I, J, & K: A more positive perception of the community and intergroup relations; respectful tone adopted between disputants  H: A more negative perception of the community and intergroup relations: some extreme actors continue to cause polarization  G, H, & I: A more positive perception of the company: a local benefactor  J & K: A more negative perception of the company: absent, with unclear motives | M & N: A more positive perception of the community and intergroup relations; respectful tone used by all but a few mine opponents  L: A more negative perception of the community and intergroup relations: mine opponents continue to cause polarization  L & M: A more positive perception of the company: a local benefactor N: A more negative perception of the company: absent, with unclear motives | O: Previous perceptions maintained, but the company’s local relationships are more prominent |
| Changes in perceptions of processes and institutions | A & B: Previous perceptions maintained, but some developments in the permit process are perceived as positive/hopeful | C, D, E, & F: Previous perceptions maintained, but some developments in the permit process are perceived as positive/hopeful | G, H, & I: A more negative perception of the permitting system: slow process and arbitrary assessments/rules; reduced political and institutional trust  J & K: Previous perceptions maintained | L, M, & N: A more negative perception of the permitting system: slow process and arbitrary assessments/rules; reduced political and institutional trust | O: A more negative perception of the permitting system: slow process and arbitrary assessments/rules; reduced political and institutional trust |
| Changes in positions and actions advocated | A & B: Previous position and preferred actions still advocated | C, D, E, & F: Previous position and preferred actions still advocated, but views on strengthened Sami rights are more prominent | G, H, & I: A more positive position: establish the mine  G, H, & I: Demands for a legislative overhaul are more prominent  J & K: A more negative position: stop the mine | L & M: Previous position and preferred actions still advocated  N: A more cautious position: “Mining if”  L, M, & N: A legislative overhaul advocated | O: Previous position and preferred actions still advocated, but primarily focused on a single mine rather than a mining district  O: A legislative overhaul advocated |
